# Socio-economic status, self-rated health and mental health: the mediation effect of social participation on early-late midlife and older adults

**DOI:** 10.1186/s13584-019-0359-8

**Published:** 2020-01-28

**Authors:** Netta Achdut, Orly Sarid

**Affiliations:** The Spitzer Department of Social Work, Ben-Gurion University of the Negev, 84105 Beer-Sheva, Israel

**Keywords:** Socio-economic status, self-rated-health, Mental health, Social participation, Information and communication technology, Structural equation models

## Abstract

**Background:**

Socioeconomic status (SES) is a major determinant of diverse health outcomes, among these are self-rated-health and mental health. Yet the mechanisms underlying the SES—health relation are not fully explored. Socioeconomic inequalities in health and mental health may form along several pathways. One is social participation which is linked to better self-rated-health and mental health. We examined (1) whether various social participation practices, including the usage of information and communication technology, relate to a unidimensional or multidimensional phenomenon (2) the relationship among SES, social participation, self-rated-health and mental health; (3) whether social participation and mental health mediates the association between SES and self-rated-health; (4) whether social participation and self-rated-health mediates the links between SES and mental health.

**Method:**

Cross-sectional data for individuals aged 35 and older were taken from the Israeli Social Survey for 2016 (*N* = 4848). Social participation practices included connection with family and friends, self-perceived-support, self-perceived trust, volunteering, civic and political involvement, and information and communication technology usage. An exploratory factor analysis was conducted for all social participation practices. We then constructed structural Equation Modeling (SEM) to explore paths of relations among SES, social participation, self-rated-health and mental health.

**Results:**

We found disparities in self-rated health and mental health across SES. Social participation practice, ‘frequency of meeting with friends’, mediated the links between SES-self-rated health and SES-mental health. Formal social participation practices along with internet usage mediated the SES- self-rated health link. Informal social participation practices and self-perceived trust mediated the SES-mental health link. Mental health mediated the SES- self-rated health link and self-rated health mediated the SES-mental health link.

**Conclusion:**

The links between SES and the two health constructs were enhanced by common and distinct social participation practices. Enhancement of social participation practices among low SES individuals is recommended. Social participation should be a prominent aspect of preventive medicine practice and health promotion interventions. Policy makers are called to support such programs as an important way to promote public health.

## Introduction

Socioeconomic inequalities in health are an important topic in social sciences and public health. Since the Black Report of 1980, which established the structural explanation for health inequality over competing explanations [1], considerable effort has been invested to understand what causes these disparities, so as to be better able to identify measures to reduce them [2]. According to the structural explanation, health inequalities are the unjust differences in health between groups of people occupying different positions in society. That is, differences in the socioeconomic circumstances of social groups along the life-course cause differences in health outcomes. Structural theorists view competing explanations for health disparities as mechanisms linking structural determinants and health outcomes. Accordingly, the Black Report claimed that the causal explanation of health disparities is rooted in socioeconomic inequalities [2, 3].

Socioeconomic inequalities in health and mental health may form along several pathways [4]. One is social participation (SP), which is broadly defined as the individual’s involvement in activities that include interactions with others in the society [5] and embraces various practices of informal and formal social connections and activities [6]. SP may be beneficial to health due to the support function provided to individuals by social networks through access to information, knowledge and to material and psychological resources that can be used to cope in times of need. SP also provides a platform for fulfilling an individual’s needs for the social interaction and integration necessary for well-being.

Many previous studies that focused on the link between SP and health measures relied on samples of old adults [7–9]. Only few focused on younger ages or examined the mediating role of different SP practices in the SES-health association [10, 11]. Based on a wide range of SP practices, including information and communication technology (ICT), the current study investigated the mediating role of SP on the SES—self-rated health (SRH) relationship and the mediating role of SP in the SES—mental health (MH) connection in individuals aged 35 and above. In line with the Black Report’s statement that differences in SES are the root cause of health disparities across the life span, it is important to better understand the factors potentially underlying health inequality in the general population. Few previous studies on this issue have taken this approach, including in their analysis both midlife and older adults [6, 12].

### Theoretical framework

Social participation, previously conceptualized as social capital, refers to a broad aspect of support networks and the relationships within and between units such as family, social groups and neighborhood [13]. Putnam [14] argues that “the core idea of social capital is that social networks have a value. Social contacts affect the productivity of individuals and groups” (pp. 18–19). Social capital can thus be defined as a personal resource accessed through social relationships [6]. However, social capital is a complex construct with distinct components [14].

Berkman et al. [15] suggested a pathways model in which social structural conditions, such as SES and poverty, condition the extent, shape and nature of social networks, such as the characteristics of network ties. These ties, in turn, provide opportunities for social support, social engagement, and access to resources such as material goods and information. The benefits and gains from social networks eventually influence an individual’s health. Berkman thus implies that individuals with low SES have weaker social networks than individuals with high SES and a lower degree of social participation. Possibly, low SES individuals struggle to provide for themselves and are less available to take part in various SP practices. Similarly, their peers and families are often struggling with their own daily needs and are not available to provide them with material, instrumental, or mental support [16]. That is, individuals of low SES are at a higher risk of experiencing weaker social support, a lower degree of a sense of belonging, and a limited access to information, whereas individuals of higher SES have greater resources and gains. Thus, SP may serve as an underlying mechanism in the SES – health link.

SP practices must, however, be differentiated. Berkman et al. [15] imply that not all ties are supportive and that the type, frequency, intensity, and extent of support provided vary across different social networks. Different SP practices involve diverse degrees of involvement and closeness and, hence, offer diverse rewards, some providing several types of support while other forms provide only one type or other human needs. For example, connections with family and friends are an important source of emotional and instrumental support. Other practices relating to social interactions outside the family include volunteer activity, membership in organizations and political involvement. They tend to build up civic skills, create an ‘equivalent status and power’ among their members and provide benefits such as information exchange and group identity. These SP practices are also called ‘opportunity-based mechanisms’ [6].

More recent conceptualization refers to SP practices by formal and informal social ties: formal SP comprises rule-bound networks whereas, informal SP is characterized by casual contacts with family and friends [6, 17].

The various SP practices might not uniformly affect health [18] let alone different construct of health, as SRH and MH. In the digital age, Information and Communication Technology (ICT) is a central platform through which social connections are made and communities built. Digital platforms are not limited to social networks but also serve other important aspects of human life, such as seeking and exchanging information, improving job matching, obtaining services from government (e-government) more efficiently, including health-care services [19], sharing health-related insights online, appropriateness of health service use [20] and easier take-up of social rights, such as social security benefits. Digital skills and actual usage are thus essential for participation in the digital economy and society. Services in the areas of health care and e-government, which are increasingly reliant on digitalized systems, may have a great effect on an individual’s health and wellbeing [19].

Internet use may also reinforce offline relationships through computer-mediated communication and may increase offline contact and social capital [19]. On the other hand, the internet exposes people to new health risks by providing inaccurate or misleading information, by online reinforcement of pathologies, and by increasing confusion due to information overload [21, 22]. Intense internet use may also shift social interactions from the real to the virtual world, replacing social activity and strong ties [19].

### Previous studies

SES is a major determinant of diverse health outcomes around the globe and across ages [23–26]. Previous studies showed that low socioeconomic status (SES) is related to higher mortality [27], poor self-rated health (SRH) and mental health (MH) [23, 25, 26, 28], higher prevalence of obesity [28] and depression [24, 29].

A large body of empirical literature documents the contribution of SP to various health measures. Overall, a positive effect of SP on different health measures was reported, including reduced mortality [30], depression, loneliness and enhanced wellbeing [9, 11, 29, 31] and SRH [9, 32, 33]. Based on longitudinal data Giordano, Björk & Lindström [34] examined the effect on SRH of social trust, frequency of meeting with friends, frequency of talking with neighbors, and being active in voluntary community groups, organizations or leisure group activities. Low levels of trust and talking less with neighbors preceded a gradual change from a good SRH to poor SRH baseline. These factors, along with the frequency of meeting with friends, also predicted improvement of SRH over time. Windsor et al. [35] employed four measures of social network structure relating to connection with family and friends (number, frequency of meeting, and duration of contact when meeting) along with participation in organized group activities. They also measured the social network quality by frequency of positive exchanges (expressions of care and interest) and negative exchanges (demands, criticisms, and arguments/tensions) in the domains of family and friend relationships. Participants with more diverse networks reported better mental health, showing direct associations of positive exchanges with better mental health, and negative exchanges with worse mental health. Using similar measures of SP, excluding family relations, Vogelsang [18] found that some aspects of SP are important for SRH, among them church or worship, meeting with friends, and charity and welfare activities. Other studies demonstrated that the link between SP and health measures varies with the form of SP and across different subgroups like gender, age and rural—urban contexts [6, 11, 18, 36]. We assume that the gains from distinct SP practices differ by the degree and type of support and that the effect of distinct SP practices on health measures will also vary.

Evidence relating Internet usage to health is quite mixed. ICT usage has generally been found to be beneficial for various health outcomes among older adults, but less positive for adolescents and young adults [37–39]. For example, Internet use is associated with higher perceived health, reduced stress, less physical illness, and lower use of health services among adults independent of socio-demographic covariates [20, 40]. However, in some studies this significant relationship disappeared once social class was considered [41]. Internet use was also positively associated with mental well-being among adults and older adults and with reduced probability of depression [42]. The recent OECD study reported that countries with a high level of internet use showed fewer reports of loneliness and Internet usage was positively correlated with general life satisfaction [19]. Yet, other studies found negative effects of Internet use, particularly the use of social networking sites, on various psychological well-being measures among adults [43, 44].

#### The mediating role of social participation in the SES-health link

Most studies have considered the direct effects of SES [25, 45, 46], SP (9, 34, 45, 46), and ICT usage [20, 38] on SRH and MH. Only few studies have examined the mediating role of SP practices on the relationship between SES and health. These studies employed single or few measures of SP as mediators, such as social trust [47] and family social capital [48]. Niedzwiedz et al. [11] showed that SP, measured by participation in external social activities (voluntary/charity, education and training courses, political or community organization) moderated the association between household wealth and loneliness among older adults. That is, participation in external social activities acts as a buffer against the adverse effects of socioeconomic disadvantage on loneliness. Rözer and Volker [47] found that social trust mediated the relation between income inequality and SRH, whereas other studies found no such effect [4]. But, as far as we know, no study to date has examined the role of the various SP practices suggested here as intermediary factors in the connection between SES-SRH and SES-MH. We therefore explored the differential mediation effect of SP practices on the links between SES-SRH and SES-MH, controlling each measure of health in turn.

*Our first aim* here was to assess whether the various SP practices including ICT usage relate to a unidimensional world of content or reflect a multidimensional phenomenon. *Our second aim* was to test the relation among SES, SP, SRH and MH. We hypothesize that individuals of low SES will demonstrate lower SP and poorer SRH and MH.

Since several researchers have demonstrated the direct positive effect of SRH and other objective health measures on MH [35, 46, 49, 50], *our third aim* was to assess the contribution of MH on SES-SRH and the role of SRH on SES-MH. We hypothesize that MH mediates the SES-SRH link and that SRH mediates the SES-MH link. Our *fourth aim* was to examine which SP practices mediate the association between SES-SRH and which the association between SES-MH. We assumed that the SES-SRH and SES-MH links are mediated by common and distinct SP practices.

## Method

### Source of data

Cross sectional data were drawn from the Israeli Social Survey (ISS) for the year 2016 (Public-Use-File) conducted by the Israeli Central Bureau of Statistics (ICBS) [51]. The ISS comprises a representative sample of 7500 persons aged 20 and older of the permanent non-institutional population of Israel, as well as residents of non-custodial institutions. Inclusion criteria in this study was individuals aged 35 and older, which yielded a total of 4848 respondents.

Self-report questionnaires, based on Blaise software developed by Statistics Netherlands [52] were administered by ICBS interviewers using laptops. The interviews were conducted in Hebrew, Arabic and Russian and each lasted about an hour [51].

### Measurement

#### Dependent variables

*Self-rated health (SRH)* was measured by three items: *(1)* general health condition: “How is your health, overall?” The response options lay on a four-point Likert scale (very good (=0); good (=1); not so good (=2); not good at all (=3)); *(2)* having any health or physical problem that interfered with daily functioning. Respondent were classified to three categories: no health or physical problem (=0); health problem that did not interfere so much or did not interfere at all with daily functioning (=1); health problem that greatly interfere or interfered with daily functioning (=2). *(3)* Had a specific health problem that might interfere with the daily function, such as *a.* walking or climbing stairs; *b.* getting dressed or bathing; *c.* carrying out household activities; *d.* problems with memory or concentration; *e.* vision problems; *f.* difficulty hearing. Responses were arrayed on a four-point Likert scale (no difficulty (=0) to complete incapacity (=3)). The response options were than dichotomized to reflect no or slight difficulty (=0) and significant or complete incapacity (=1). These six dichotomized variables were summed up to an overall index, ranging from 0 to 6. The index thus indicated the number of limitations that interfered with daily functioning. Finally, using the sum of the final scores in the three items detailed above, we created an overall physical health scale, ranging from 0 to 11. We recoded the scale so that higher score reflected better self-rated health condition. Reliability analysis (Cronbach’s α) for this scale was not calculated. Following Taber [53] we did not assume that our measurement of health was unidimensional or that individuals who reported being in poor or bad health would suffer from several health problems. For example, an individual with memory or concentration limitation is not necessarily assumed to have additional health problems [53].

*Mental health (MH)* was assessed on six questions: *(1)* Do you ever feel lonely (frequently, sometimes, seldom, never)? *(2)* Have you felt pressured in the past 12 months? *(3)* Have you felt depressed in the past 12 months? *(4)* Did you feel able to deal with your problems in the past 12 months? *(5)* Did you feel full of energy in the last 12 months? *(6)* Have worries prevented you from sleeping in the last 12 months? Answers to items 2–6 were set on a four-point Likert scale from always (=1) to never (=4). After reversal of the questions (4) and (5), a summed score for these six questions was calculated. The final score ranged from 6 to 24, with a higher score reflecting better mental health. Cronbach’s α = .769.

The two measures SRH and MH correlated moderately. Pearson correlation coefficients were 0.41. Despite some overlap in these variables we treated them as separate constructs, [see also 9, 23].

#### Independent variables

*Socio-Economic Status (SES)* was measured by three indicators: education level, average income per capita in the household and home ownership (no home ownership (=0); owns a home (=1)). Participants’ highest education level was recorded according to the International Standard Classification of Education [54] and divided into low (less than lower secondary education, or lower secondary education completed), medium (upper secondary education or post-secondary non-tertiary education completed) and high (tertiary education completed). Average income per capita was defined by three categories: low (up to NIS 2000/USD 530); medium (NIS 2001/USD 531 - NIS 4000/USD 1060); and high (NIS 4001/USD 1061 and above).

#### Mediating variables

*Social Participation (SP)* was divided into *informal* and *formal practices*:

1. *Informal practices* consisted of four questions: *a.* satisfaction with relations with family members (not so satisfied and not satisfied at all (=0); very satisfied or satisfied (=1)); *b.* frequency of meeting friends, or talking to them. Measured on a four-point Likert scale (less than once a month (=0); once or twice a month (=1); once or twice a week (=2); daily, or almost daily (=3)); *c.* “If you were in trouble, are there people whose help you could count on?” defined as *self-perceived support* (1 = yes); and *d.* “In general, can you trust most people or do you have to be wary of them?” defined as *perceived trust* (1 = most people can be trusted)
2. *Formal practices of SP* included: *a.* engagement in volunteer activities in the previous 12 months, in the framework of an organization or movement (=1); *b.* involvement in public or political life on a national or local level in the past 12 months (=1). Those who reported involvement in at least one activity received the value 1.

An additional domain of SP was using Information and Communication Technology (ICT), measured by nine items signifying the intensity and purposes of this activity. The first item was: “During the last three months, how many times a week did you use the internet, through a computer or a mobile phone? (0 = not at all; 1 = twice a week or less; 2 = every day or almost every day). The remaining eight items were: used the internet *a.* to search for information; *b.* for e-mail; for discussion groups and social networks, such as chat rooms, forms, WhatsApp, Twitter; *c.* for online games or download games; *d.* for banking transactions and paying bills; *e.* for viewing or downloading files, such as photos, music, movies; *f.* to purchase products or services; *g.* to obtain services from government agencies; *h.* for phone calls or video, such as skype. Answers to each of the above items were dichotomies (yes = 1). Based on the sum of these nine items we computed an ICT scale from 0 to 10, a higher score reflecting more intense ICT use. Cronbach’s α = .910.

### Participants

Table 1 summarizes the descriptive statistics of the sample. Age was fairly evenly distributed across the study population. The majority were married (74%), with 44% having at least one child aged under 18 in the household. Immigrants and the Arab respondents each comprised almost one fifth of the sample (18.9 and 18.1%, respectively). Sixty-two percent were employed, where this rate was higher among the working-age population (77.3%) (not shown). As for SES indicators, medium or high education was reported by 34.2 and 35.6% of the respondents respectively, as compared with a lower rate of respondents with low education (30.1%). About half of the respondents had high average income per capita, and more than three-quarters reported home ownership.

**Table 1:** Descriptive statistics of the study variables, Israeli social survey for 2016

	*N*	%/ Mean (SD)
Demographic		
Age group		
35–44	1377	28.4
45–54	1144	23.6
55–64	987	20.4
65 and above	1340	27.6
Male (=1)	2280	47.0
Marital status		
Single	326	6.7
Married	3600	74.3
Divorced/Separated	534	11.0
Widowed	388	8.0
Children aged 0–17 in the household (=1)	2135	44.0
Immigrant (=1)	914	18.9
Arab (=1)	876	18.1
Employed (=1)	3004	62.0
SES		
Education		
Low	1458	30.1
Medium	1725	35.6
High	1658	34.2
Average income per capita in the household		
Low	857	21.1
Medium	1204	29.6
High	2002	49.3
Home ownership (=1)	3751	77.4
Social participation		
Satisfaction with family relationships (1 = very satisfied or satisfied)	4606	95.0
Frequency of meeting/speaking with friends (0–4)		
No friends	479	9.9
Less than once a month	125	2.6
Once or twice a month	521	10.8
Once or twice a week	1815	37.6
Daily, or almost daily	1889	39.1
Self-perceived-support (yes = 1)	4371	91.3
Self-perceived-trust (yes = 1)	1925	41.2
Volunteering (=1)	951	19.6
Public/political involvement (=1)	637	13.1
ICT use scale (0–10)	4838	6.11 (4.11)
*N*	*4848*	

The great majority of respondents (95%) reported being very satisfied or satisfied with regard to family relations, about 77% stated that they met or spoke with friends once a week or more, and about 90% had people to count on in times of trouble. By contrast, when referring to self-perceived trust, only 41% believed that most people could be trusted. About 20% had taken part in volunteering activity, and the mean level of use of ICT was rather high, at 6.11 (on a 0–10 scale).

### Data analysis

For the various SES indicators, we conducted *Varimax exploratory factor analysis (EFA)* [55] using the initial Eigenvalue cutoffs to estimate the factor analysis. The factor solution yielded one factor with Eigenvalue above 1.0, comprising two indicators that loaded significantly: education level (.823), and average income per-capita in the household (.823). The cumulative variance explained by this factor was 67%. Home ownership did not correlate closely with the other components, therefore we treated it as a distinct variable.

To examine the first aim, we conducted *Varimax exploratory factor analysis (EFA)* for all forms of SP. Two factors emerged with Eigenvalue above 1.0, the first comprised of three items that loaded significantly: volunteering (.675), political involvement (.725) and use of ICT (.671); the second was satisfaction with relationships with family members (.783) and self-perceived-support (.704). The cumulative variance explained by these two factors stood at 53%. Two distinct items did not correlate with the above two factors: ‘frequency of meeting friends or talking with them’ and ‘self-perceived-trust’.

To test the second aim, we conducted bivariate analysis of SRH and MH by SES and by SP. The Pearson correlation was calculated for SES factor and SRH, for SP factors and SRH, and for ICT scale and SRH. The same was done with MH. Using ANOVA (F-test) we examined mean differences in SRH and MH by the categorical variables of education, household income, and frequency of meeting with friends. The t-test examined mean differences in SRH and MH by home ownership, volunteering, political/civic involvement, satisfaction with family relations, perceived support and perceived trust. Six hierarchical linear regression models – three for each health outcome - estimated SRH and MH (results from these estimations are presented in Additional file 1:

To investigate the third and fourth aims, we constructed two Structural Equation Models (SEM) using SPSS statistic 25 with AMOS module. Both models were drawn by placing demographic variables detailed in Table 1 (not shown in the final SEM models) and SES indicators as predictors for SRH and MH. Next, by an iterative process, for each model we added pathways that improved the model’s fit. Given a postulated association between the SP forms we also drew residual covariance paths between these variables. Adding these paths and the residual covariance paths was supported by the AMOS modification indices function. All models were estimated by the maximum likelihood estimation method. Since quality of the fit of SEM models is affected by sample size, multiple model fit indicators were assessed including χ^2^, the ratio of the χ^2^ to degrees of freedom (χ^2^ /df), the comparative fit index (CFI), the Tucker-Lewis Index (TLI), the Normed Fit Index (NFI) and a root-mean-square error of approximation (RMSEA). Ideally, for a model that fitted the data, the χ^2^ would not be significant. However, since χ^2^ is sensitive to sample size, in cases of large samples, it has been suggested to use the ratio of the χ^2^ to degree of freedom. A model demonstrates a reasonable fit if the statistic adjusted by its degrees of freedom is < 5.0 and > 2 [56, 57]. Values close to 0.95 for the CFI, TLI and NFI (higher is better), and close to 0.05 for the RMSEA (lower is better) indicate a good fit of the data to the model [58].

Bootstrapping was used to test the significance of the mediation effect. This is a non-parametric method based on resampling with replacement, which is reiterated many times within the study sample according to its original size [59]. The indirect effect is computed from each of these samples and a sampling distribution can be empirically generated. The distribution allows computing a confidence interval (CIs), which is checked to determine if zero is in the interval. If zero is not in the interval, then the researcher can be confident that the indirect effect is different from zero, i.e., statistically significant [60]. We further tested the robustness of our results on the mediation effect using the RMediation package, which also builds CIs for mediation effects based on the product of two regression coefficients and their standard error [61]. Based on the CIs we tested the significance of each specific mediation. As in bootstrapping, in cases when zero is not in the 95% confidence interval, it can be concluded that the indirect effect is significantly different from zero at *p* < .05.

## Results

### Descriptive results

Table 2 presents the mean value (SD) of SRH and MH indices by SES and SP. With respect to continuous explanatory variables, Pearson correlations are presented.

**Table 2:** SRH and MH by SES and social participation, Israeli social survey for 2016

	SRH Mean (SD)	F/ T-test/Pearson correlation	MH Mean (SD)	F/ T-test/Pearson correlation
Overall	8.23 (3.10)		18.04 (3.94)	
SES				
Education				
Low	6.89 (3.60)	231.34**	16.97 (4.45)	76.11**
Medium	8.53 (2.80)		18.45 (3.85)	
High	9.11 (2.46)		18.54 (3.35)	
Average income per capita in the household				
Low	7.16 (3.68)	119.58**	16.81 (4.48)	62.81**
Medium	7.73 (3.32)		17.85 (4.12)	
High	8.91 (2.50)		18.59 (3.42)	
Home ownership				
Yes	8.34 (3.03)	−4.51**	18.31 (3.82)	−8.68**
No	7.86 (3.31)		17.12 (4.21)	
SES Factor: education Average Income per capita in the household	–	.313**	–	.228**
Social participation				
Volunteering				
Yes	9.12 (2.39)	−11.73	18.67 (3.47)	−5.93**
No	8.02 (3.21)		17.89 (4.03)	
Public/political involvement				
Yes	8.11(3.18)	− 8.68**	18.62 (3.18)	− 4.65**
No	9.02 (2.33)		17.95 (4.04)	
ICT use scale (0–10)		.407**	.157**	
Factor 1: volunteering, political involvement, use of ICT	–	.289**	–	.125**
Satisfaction with family relationships				
Very satisfied or satisfied	8.30 (3.07)	− 5.36**	18.18 (3.84)	−9.13**
Not so satisfied or not satisfied at all	7.00 (3.41)		15.07 (4.66)	
Self-perceived-support				
Yes	8.39 (3.01)	−8.88**	18.27 (3.77)	−10.47**
No	6.79 (3.57)		15.70 (4.67)	
Factor 2: satisfaction with family relations, having people to count on in times of trouble	–	.171**	–	.221**
Frequency of meeting/talking with friends (0–4)	–	.208**	–	.174**
No friends	6.35 (3.69)	57.11**	15.93 (4.80)	42.12**
Less than once a month	7.45 (3.43)		17.86 (4.01)	
Once or twice a month	8.03 (2.99)		17.60 (4.04)	
Once or twice a week	8.39 (3.04)		18.27 (3.72)	
Daily, or almost daily	8.66 (2.81)		18.46 (3.71)	
Self-perceived-trust				
Most people you can trust	8.85 (2.72)	−10.85**	18.76 (3.47)	−10.432**
You have to be wary of people	7.87 (3.25)		17.54 (4.14)	

*p < .05; **p < .01

The top panel of Table 2 shows the mean value of SRH (8.23 + 3.10) and MH (18.04+ 3.94) in the overall sample, indicating relatively high values. Both SRH and MH mean values differed on all independent variables and indices. As expected, a positive correlation was found between the SES factor to SRH and MH, and between home ownership to SRH and MH. SP was associated with better SRH and MH: factor 1 (volunteering, political and ICT use) correlated with SRH, whereas factor 2 (‘satisfaction with family relations’^1^ and ‘having people to count on’) correlated with MH.

### SEM models

**Fig. 1:**
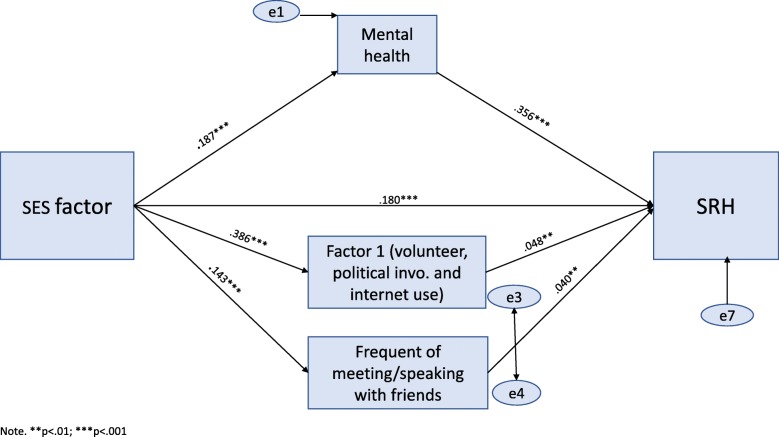
Path analysis model showing the direct and indirect effects of SES on SRH, with mental health and social participation as mediators

**Fig. 2:**
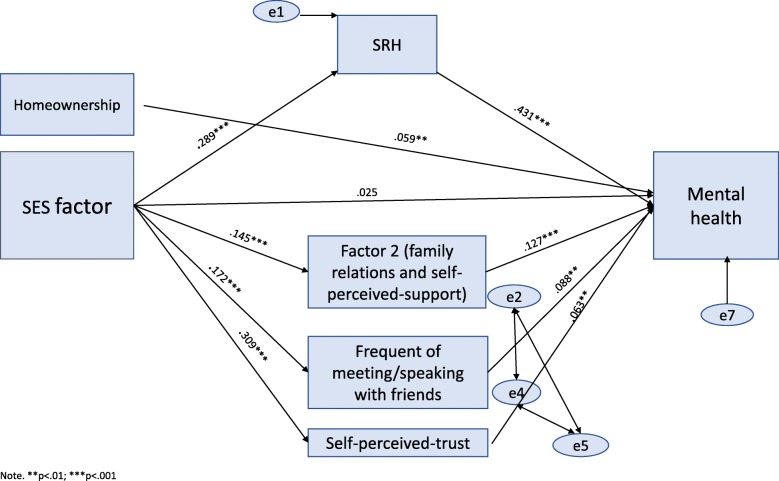
Path analysis model showing the direct and indirect effects of SES on mental health, with SRH and social participation as mediators

Figures 1 and 2 present two estimated SEM models for SRH and MH, respectively. Standardized coefficients, based on bootstrapping, are displayed on the figures. Additionally, Additional file 1 includes full regression results based on OLS estimations for SRH and MH. We present only the mediation effects that were statistically significant based on the RMediation test detailed in data analysis section. SRH (Fig. 1): the χ^2^ value was significant in this model (χ^2^ (15) = 58.02, *p* > 0.05) due to the large sample size (*n* = 4848). However, this model gave a very good fit in all other indices (χ^2^ /df = 3.41, RMSEA = .024, CFI = .995, TLI = .984, NFI = .993), and explained 42.2% of SRH variances. One direct pathway linked the SES factor to SRH (standardized regression weights β = .18, *p* > 0.01). Three mediating pathways appear in the model linking SES to SRH: in the first mediating pathway, MH mediated the links between SES to SRH. To elaborate, higher SES led to better MH (β = .19, *p* > 0.01) which in turn led to better SRH (β = .36, *p* < 0.001). The second indirect pathway indicated that the formal SP factor of volunteering, political and civic involvement and ICT mediated the links between SES and better SRH. That is, higher SES produced a higher score in this factor (β = .386, *p* < 0.001), which led to better SRH (β = .50, *p* < 0.01). In the third mediating pathway, ‘frequency of meeting friends’ served as a mediator between SES to SRH: SES impacted friends’ relations (β = .14, *p* < 0.001) and friends’ relations enhanced SRH (β = .04, *p* < 0.01).

MH (Fig. 2): the χ^2^ value was significant in this model (χ^2^ (18) = 55.37, *p* > 0.05). However, this model gave a very good fit for all other indices (χ^2^ /df = 3.07, RMSEA = .021, CFI = .995, TLI = .983, NFI = .993), and addressed 30.0% of MH variance. Direct pathways linked home ownership with MH (β = .06, *p* < 0.05), but no direct pathway was found between SES and MH (β = .025, n.s). Results from OLS regressions showed that once SP indicators were added as predictor, the SES coefficient became insignificant in explaining MH (see Additional file 1: , model 4–6). This suggests that there was full mediation. Fig. 2 reveals four pathways from SES to MH. In one pathway SRH mediated the links between SES and MH. In another pathway the SP practice of ‘family relations and self-perceived-support’ mediated the links between SES and MH. The SES-MH pathway was also mediated by self-perceived-trust. In another pathway SES-MH was mediated by the practice ‘frequency of meeting friends’.

## Discussion

Since the 1980 Black Report, establishing the structural explanation for health inequalities [1], considerable effort has been exerted to understand what causes this inequality, so as to be better able to identify measures to reduce it [2]. In this study we proposed an optional pathway to the SES-health link through a measure of diverse SP practices along with Internet use.

Our findings confirm that SP is a multidimensional construct [14, 18] and are supported, to some extent, by the conceptualization of SP practices as formal and informal forms. Informal SP forms comprise family relations and perceived support, whereas formal SP practices include volunteering, political and civic involvement and ICT usage. The ‘frequency of meeting friends’ and perceived trust were distinct SP practices that did not align with the formal or informal forms of SP. Although ICT usage potentially embraces informal practices such as casual connection with close and remote friends and relatives through Facebook, WhatsApp or other social networks, it can be used as a platform for formal practices through membership in organizations, activity in social and civic movements and community activity through a neighborhood Facebook page. ICT, as measured here, refers also to uses other than SP that have proven health benefit [19, 20]. For example, information searches and the use of e-services facilitate accumulation of knowledge and access to health services and treatment options. Either way, the classification of Internet use as part of formal SP practices needs to be further examined in future studies.

As we hypothesized, we found disparities in SRH and MH across SES. These findings here fit the accumulated knowledge on the relations between SES-SRH and SES-MH [23–25]. Here SRH was a predictor of MH and vice versa as shown by the OLS estimations. We confirm previous studies showing that MH and others aspects of well-being, such as happiness, enjoyment and optimism, were positively related to SRH and physical health and negatively related to disease and mortality [35, 46, 49, 50]. Furthermore, as shown in our SEM models, better MH mediated the SES-SRH association, whereas SRH mediated the SES-MH link. These findings, along with those from other studies, demonstrate that health and mental health are two interrelated but different phenomena which have both common risk factors and distinct resilience factors [62, 63].

Finally, we examined the distinct mediating role of SP forms on SES-SRH and SES-MH assuming that SP is a multidimensional concept with different intensities of effects on health, as well as different constructs of health. Higher SES was associated with stronger SP, which, in turn, was positively related to both SRH and MH. To elaborate, health inequality was partly related to a lesser degree of both formal and informal SP practices. Low SES individuals may engage less in the various forms of social participation or benefit less from these interactions and activities, which might result in a worse health outcome. As we assumed, variation in SP mediators were found across the two health constructs. SRH was enhanced by formal SP forms including Internet use practice while the SES-MH link was mediated by informal SP forms and self-perceived-trust. Our results demonstrate that different SP gains are important in enhancing different health outcomes. A possible explanation for the mediating role of formal SP practices and ICT use in the SES-SRH link is that these practices enable individuals to access information, gather knowledge and power and exert a certain degree of control over their physical health. In the SES-MH link informal SP was important. Having social trust, a sense of support and a supportive network of family ties may imply close and intimate relationships that benefit mental health [15]. Furthermore, mediation effects also varied in intensity across the models: While informal SP practices, frequency of meeting with friends and trust fully mediated the SES-MH link, formal SP practices and frequency of meeting with friends only partially mediated the SES-SRH link. This may mean that “physical health”, more than MH, is influenced by “real” socio-economic circumstances, such as limited access to healthcare and insufficient nutrition. It also may imply that alternative underlying mechanisms are important in the SES-SRH link, such as risk behaviors and macro-level mechanisms, including the characteristics and the quality of the health-care system and public health expenditure. However, keeping in mind that SP practices do not uniformly affect SRH and MH, it is important to look for practices that can enhance both SRH and MH. Such practices can serve as a first line protective mechanism in enhancing diverse health constructs. In our SEM models a significant mediator was frequency of meeting with friends and thus regularity and constancy of meeting friends may be important in enhancing both SRH and MH. Other regular practices that relate to better health are daily habits, and frequency of meeting friends can be seen as a habit showing recurrence and repetition important in promoting health.

This study has several limitations. Given its cross-sectional design, our attributions of causality must be taken with caution. Although our explanations are rooted in theoretical models and research, and the SEMs suggest causality, alternative explanations cannot be ruled out, and plausible alternative models may fit the data as well or better than the models we tested. Our aim in this analysis was to help develop knowledge by examining potential underlying mechanism of the SES-health link, an approach used by previous health studies also in examining potential mediators in the SES-health link [4, 23].

Since our tests show that SP mediators are significant, they provide a strong rationale for testing the causal link through longitudinal data or experimental designs. Thus, we recommend a big data base to adapt a longitudinal prospective design and to follow the same individuals across time. Furthermore, given the complexity of our model and analyses, we did not test alternative path directions (health-to-SES or SP-to-SES) or subgroup differences by age or ethno-culture groups. Future studies should address these issues. Finally, important mediators such as financial strain and deprivation indices should also be taken into account in exploring the paths between SES and health.

## Conclusions and recommendations

Implication of this study are relevant for policy makers as well as for health and social services practitioners. The enhancement of SP practices should be considered as a way to promote health—in older adults, but also in early-late midlife adults. Particularly among lower SES individuals, enhancement of formal practices of SP, such as teaching ICT use, encouraging volunteering and community activities may improve their SRH. Practitioners working in the community and in mental health services are encouraged to collaborate and construct interventions to enhance perceived trust and informal SP forms, especially in low SES groups as these can act as buffers in the SES-MH association. Moreover, considering the aims of an intervention and the target population, interventions should place different emphases on specific SP practices. For example, while, frequent meetings with friends should be a central aspect in an SP intervention that aims to enhance both SRH and MH, enhancing perceived trust should be a central aspect in the field of MH. Finally, as suggested by both mediation models, interventions to promote MH may help to moderate SES’s negative effect on SRH and vice versa. Policy makers should support programs enhancing SP as an important means of promoting public health, regardless of the need to deal with the root causes of health inequality in society, i.e., socioeconomic disparities.

## Supplementary information

**Additional file 1:** OLS Regression: prediction of SRH and mental health.

## Data Availability

The data sets analyzed during the current study are available from the Israel Central Bureau of Statistics upon request.

## Abbreviations

ICT: Information and communication technology
MH: Mental health
SES: Socioeconomic status
SP: Social participation
SRH: Self-rated-health

## Footnotes

1 No substantial variance was found in the variable satisfaction with family relationship (see Table 1). However, this measure is part of the factor score of informal SP. Being part of a total factor score somewhat reduces this problem.
