# Supplementary material for: Socio-economic status, self-rated health and mental health: the mediation effect of social participation on early-late midlife and older adults
Source: Isr J Health Policy Res. 2020 Jan 28;9:4. doi: 10.1186/s13584-019-0359-8 (PMC6988248; doi:10.1186/s13584-019-0359-8)
Supplement: Supplementary file 1 — Additional file 1: OLS Regression: prediction of SRH and mental health. [file 13584_2019_359_MOESM1_ESM.docx]

**Appendix A**

Table A shows the results of six hierarchical linear regression models. In models 1–3, SRH was the dependent variable, whereas in models 4-6 mental health was the dependent variable. Coefficients (standard errors in parenthesis) of the independent variables are presented. All models control for demographic characteristic and employment status (not shown).

Starting with SRH, SES proved a strong predictor of SRH. In models 1-3 it can be seen that higher SES in terms of education and average income per capita of the household correlated positively with SRH. Home ownership was an insignificant coefficient in the first model, and was significantly negative in the second and third models. These associations are not in the expected direction.

Mental health was positively correlated with SRH, and in the second model makes a distinct contribution of 12.2% to the total explained variance. The third model showed that both social participation factors, along with the frequency of meeting and talking with friend, had a positive effect on SRH. However, this step made only a slight distinct contribution to the total explained variance (.03%). Our findings are in line with previous studies indicating that both mental health and social participation promote SRH.

With respect to mental health, in the first two models (4 and 5) a higher SES factor score was correlated with better mental health. Home ownership was strongly and positively related to mental health across all models (4 to 6). SRH was positively correlated with mental health, and made a distinct contribution of 16.2% to the total explained variance in model 5. Finally, model 6 indicated that factor 2 (family relations and having people to count on), frequent meetings/speaking with friends, and self-perceived-trust promoted mental health.

The findings here demonstrate variations in the contribution of SES and social participation across the two health outcomes. First, in all models the total explained variance is much higher for SRH than for mental health. The most pronounced difference is observed in the prediction power of the first model – containing demographics and SES. In addition, the regression analyses present variation across the different measures of SES and social participation. For example, home ownership and self-perceived-trust are predictors of mental health whereas they have no effect on SRH. Social participation factor 1 (volunteering, civic/political involvement, ICT) was a significant predictor of SRH only. We also observed substantial change in the coefficients of the SES factor across the models: for both health measure the coefficients are highest in the first model (model 1 and 4 respectively) and reduces when the other health measure insert (MH in model 2 and SRH in model 5), and further reduces when SP is inserted (model 3 and 6), implying for potential mediation effect of SP in the SES-health links. The SES factor became insignificant for MH when adding SP (model 6), suggesting that full mediation occurred.

Table A. OLS regressions: Prediction of SRH and mental health^(a)^

|  | SRH |  |  | Mental Health |  |  |
| --- | --- | --- | --- | --- | --- | --- |
|  | Model 1 | Model 2 | Model 3 | Model 4 | Model 5 | Model 6 |
| **SES (2 domains)** |  |  |  |  |  |  |
| **SES Factor:** education,  average income per capita in the household | .660**  (.051) | .480**  (.046) | .413**  (.050) | .611**  (.075) | .196**  (.069) | .064  (.073) |
| Home ownership (=1) | .160  (.112) | -.300*  (.101) | -.252*  (.102) | .476**  (.164) | .577**  (.148) | .636**  (.147) |
| **Mental health** | -------- | .295 **  (.010) | .285**  (.010) | -------- | -------- | -------- |
| **Self-rated-health** | -------- | -------- | -------- |  | .630**  (.021) | .595**  (.021) |
| **Social participation (four domains)** |  |  |  |  |  |  |
| **Factor 1:** volunteering, political involvement, use of ICT | -------- | -------- | .089*  (.044) | -------- | -------- | -.071  (.064) |
| **Factor 2:** satisfaction with family relations, having people to count on in times of trouble | -------- | -------- | .099*  (.039) | -------- | -------- | .401**  (.056) |
| Frequent meetings/talking with friends (0-4) | -------- | -------- | .064*  (.032) | -------- | -------- | .284**  (.046) |
| Self-perceived trust (1= most people you can trust) | -------- | -------- | .074  (.079) | -------- | -------- | .493**  (.114) |
| R^2^ | .341 | .463 | .466 | .126 | .288 | .311 |
| R Square Change |  | .122** | .003* |  | .162** | .023** |

Note. *p<.05; **p<.01 ^(a)^ All models control for age; gender; marital status; children aged 0-17 in the household; immigrants; Arabs; employment status.
